# Molecular Mechanisms Contributing to Mesenchymal Stromal Cell Aging

**DOI:** 10.3390/biom10020340

**Published:** 2020-02-21

**Authors:** Simona Neri, Rosa Maria Borzì

**Affiliations:** IRCCS Istituto Ortopedico Rizzoli, Laboratorio di Immunoreumatologia e Rigenerazione Tissutale, 40136 Bologna, Italy; rosamaria.borzi@ior.it

**Keywords:** mesenchymal stem/stromal cells (MSC), MSC senescence, in vivo aging, in vitro aging, rejuvenating strategies

## Abstract

Mesenchymal stem/stromal cells (MSCs) are a reservoir for tissue homeostasis and repair that age during organismal aging. Beside the fundamental in vivo role of MSCs, they have also emerged in the last years as extremely promising therapeutic agents for a wide variety of clinical conditions. MSC use frequently requires in vitro expansion, thus exposing cells to replicative senescence. Aging of MSCs (both in vivo and in vitro) can affect not only their replicative potential, but also their properties, like immunomodulation and secretory profile, thus possibly compromising their therapeutic effect. It is therefore of critical importance to unveil the underlying mechanisms of MSC senescence and to define shared methods to assess MSC aging status. The present review will focus on current scientific knowledge about MSC aging mechanisms, control and effects, including possible anti-aging treatments.

## 1. Introduction

Mesenchymal stem/stromal cells (MSCs) or adult stem cells are tissue-specific pools of progenitor cells with long-term self-renewal ability and differentiation potential, with a fundamental role in tissue and organ homeostasis [1,2,3,4,5,6]. These cells possess both differentiation plasticity (stemness) and tissue supportive functions (stromalness) that can coexist and overlap, with differences depending on tissue source, donor characteristics, culture conditions and delivery strategies, leading to alternative best fittings for the term “stem” or “stromal” [7]. MSCs represent a lifelong reservoir for the generation of somatic cells and for cell replacement. They emerged in the last years as powerful tools in regenerative medicine and tissue engineering, mostly for their easy of isolation and expansion, for their immunomodulatory [8,9,10,11] and paracrine activities, exerted through the release of soluble factors [12], exosomes and microvesicles [13], thus modulating tissue microenvironment, counteracting inflammation and stimulating repair [2,8,14].

During aging of the organism, MSCs also age, and this implies an impairment of stem cell functions contributing to the progressive decrease in tissue maintenance and repair, a characteristic of the aging process. Actually, stem cell exhaustion is considered one of the promoters of aging. One example is the delay in fracture healing with advanced age, attributed to a decreased number and function of MSCs [15]. 

Due to the low abundance of MSCs in human adult tissues (about 1/10^6^ cells in adult bone marrow and 1/10^3–4^ cells in adipose tissue and umbilical cord) [16], frequently ex-vivo expansion precedes therapeutic administration, to obtain a clinically relevant number of cells. However, in vitro expansion drives senescence and reduces DNA synthesis and repair efficiency, finally leading to DNA damage accumulation [2,17,18,19,20,21,22,23,24]. Persistent DNA damage can block transcription and replication and drives the aging process [25].

The most relevant effect of in vitro expansion is progression to replicative senescence [21,22,24] accompanied by morphological and functional alterations, and modification of the secretory phenotype. With aging, MSCs show reduced immunomodulation [26,27], proliferation, differentiation potential and homing ability [28,29,30,31]. They develop the so-called senescence-associated secretory phenotype, SASP [21,24,32,33], and modify their transcriptional profile [34]. All these modifications can impair MSC functional properties and are critical for the efficacy and safety of MSC treatments, since they can cause not only therapeutic failure, but also unwanted effects, such as the exacerbation of the inflammatory response and the tumor-promoting activity mediated by the SASP, thus raising a safety issue [2]. It appears therefore of critical importance to evaluate the impact of senescence on MSC biological properties and to monitor senescence of MSC preparations before application [35].

The aim of the present review is to provide the reader with the current knowledge about MSC aging, acquired both in vivo and/or in vitro, and about the effect of aging on the biological properties of these cells, finally affecting their therapeutic potential. Possible techniques to limit or control cell aging will be discussed as well.

## 2. Cellular Senescence

Senescence is a cellular response to endogenous and exogenous stresses limiting proliferation of damaged and aged cells [36,37,38]. It can be induced by harmful stimuli such as DNA damage, telomere shortening, oncogenic insults, metabolic stress, epigenetic changes, and mitochondrial dysfunction. Senescence is involved not only in pathological processes like tumor progression/promotion and tissue repair, but also in physiological mechanisms like aging, development and tissue homeostasis. Senescent cells accumulate with aging in the different tissues of the organism. Two types of senescence can be distinguished: acute senescence, induced by extrinsic stressors, and chronic senescence, induced by prolonged damaging stimuli, such as replicative stress and the related DNA damage [39]. Senescence is a dynamic process, progressive and multistep, irreversible at the end and completely different from a quiescent state (that is reversible) [40]. Progression from early (reversible) to full (irreversible) senescence implies extensive chromatin remodeling and the production of a senescent secretome.

Senescence includes a stable growth arrest flanked by both genetic and epigenetic changes, chromatin remodeling, mitochondrial alterations [41], increased autophagy markers but decreased autophagic flux, and a modified secretome [42]. The senescent phenotype is characterized by an enlarged and flat cell morphology, an increase in SA-β-galactosidase (SA-β-gal) activity, DNA damage, and modified gene expression. One of the typical characteristics of senescent cells is a massive alteration of the genome architecture with specific phenotypes reflecting the different stress responses that induced senescence [43].

Growth arrest, the main characteristic of senescence, is a way to avoid replication of damaged or transformed cells and is sustained by the p16INK4a/Rb and p53/p21CIp1 pathways [36]. The accumulation of damage to DNA, proteins, lipids and carbohydrates is considered as responsible for the functional decline occurring during cell senescence. Telomere attrition is one of the insults that can induce senescence. It can be due to replicative stress (normal cells do not express telomerase, therefore they progressively shorten telomeres at each cell division until a critical size is reached, activating a DNA damage response) or to environmental stress [26].

A central role in senescence development is played by epigenetics. DNA methylation regulates gene activity and somehow triggers the process of aging. Actually, in vitro replicative senescence and in vivo aging are both characterized by epigenetic modifications. It is estimated that approximately 60% of the genomic CpG sites become hypomethylated and 40% hypermethylated upon aging [44]. Interestingly, senescence is characterized by site specific changes in the DNA methylation pattern [45], even if the mechanisms that govern these modifications are still largely unknown [46]. These methylation signatures represent “epigenetic clocks” emerging as useful tools to estimate donor age or to check cell preparations, even if epigenetic clocks are less precise for cell cultures, due to their heterogeneity [46]. In general, despite some overlaps, replicative senescence and aging display independent methylation patterns (reviewed by [46]). Notably, cellular reprogramming into induced pluripotent stem cells (iPSCs) is able to completely reverse age-associated DNA-methylation patterns thus rejuvenating cells [46,47].

Senescent cells modify their secretome and produce the so-called SASP that sustains and amplifies senescence through an autocrine/paracrine regulatory loop involving SASP-producing and surrounding cells. Thus, the SASP is responsible for a transmission of senescence to adjacent cells [48] and for persistent chronic inflammation (defined as inflammaging) [49]. The SASP presents a dynamic composition including pro-inflammatory cytokines (such as interleukins IL-1α, IL-1β, IL-6 and IL-8), chemokines, growth factors and proteases (such as metalloproteinases MMP1, MMP3 and MMP10) whose combination is dependent on cell type, microenvironment and on the mechanism of senescence induction [36]. Among the most important SASP regulators there are C/ebpβ (CCAAT/enhancer-binding protein β), NOTCH1 (Notch homolog 1), NF-κB (nuclear factor κB), mTOR (mammalian target of rapamycin), the transcription factor GATA4 (GATA binding protein 4) [36,48,50]. In addition, metabolic dysfunction, involving mTOR and insulin pathways [51,52], can regulate cell secretome. The secretory phenotype of senescent cells seems to undergo a time-dependent two-wave composition driven by the dynamic activity of NOTCH1, directing two distinct senescence phenotypes: a first pro-senescent TGF-β1-dependent secretome, followed by a second pro-inflammatory secretome [40,53].

Senescent cells are also characterized by important changes in mitochondrial function. Mitochondrial dysfunction has been shown to contribute to senescence and to SASP production [41]. Based on the “free radical theory of aging”, reactive oxygen species (ROS) are the main responsible for age-related loss of cellular functions and aging. In this scenario, a fundamental role in the response to oxidative stress is played by TRX (thioredoxin-1). TXNIP (thioredoxin-interacting protein), a negative regulator of TRX, influences the aging process and accumulates with aging, resulting in decreased oxidative stress resistance [54].

Autophagy is another fundamental player in cellular senescence. It is a constitutive catabolic pathway involving lysosomal self-degradation of damaged cellular components, like proteins and mitochondria, recycled after degradation in order to maintain cellular homeostasis. In basal conditions the process occurs at very low levels, but it is activated following cellular exposure to stress insults to promote cell survival [55].

## 3. MSC Senescence

MSC senescence represents a relevant driver of organismal aging and contributes to the pathogenesis of age-related diseases. Accordingly, senescent cells accumulate in aged tissues [56]. Key regulators of MSC aging are still incompletely identified and can represent therapeutic targets to counteract age-associated diseases and organismal aging. MSC senescence can arise from different stimuli, including oxidative stress, irradiation, chemicals (inducing acute senescence) or replicative exhaustion (inducing chronic senescence) [39].

**Figure 1:**
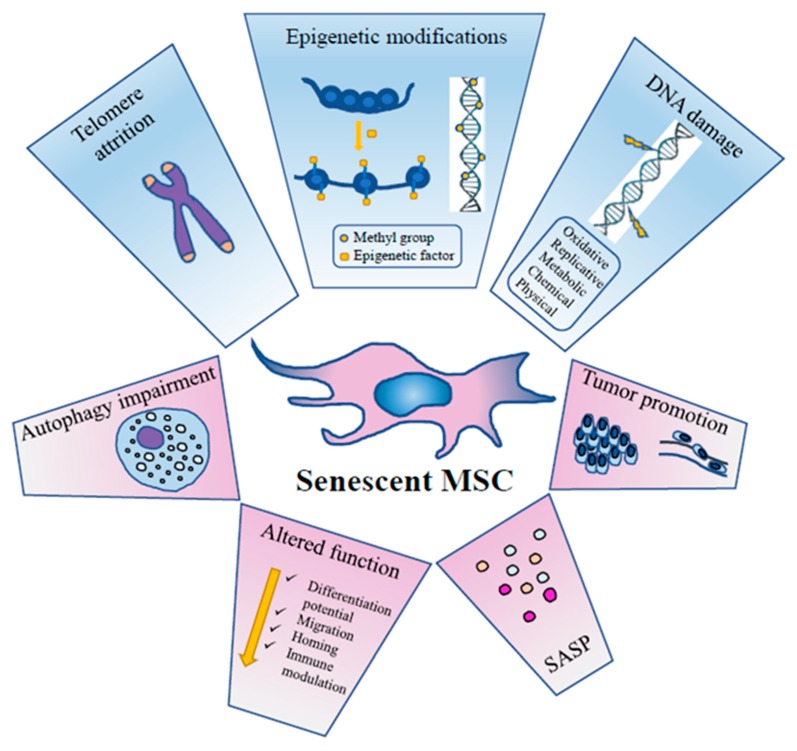
The principal events inducing MSC senescence and their effects on MSC activity, metabolism and function.

As for other cellular types, aging of MSCs is a dynamic process taking place through a series of steps that follow the initial growth decline [31,57]. It is accompanied by functional alterations due to metabolic, genetic, epigenetic, transcriptional and translational changes [31,58,59,60] (Figure 1). 

Senescent MSCs loose metabolic flexibility, they show an impairment of the autophagic flux with reduced active vacuoles, and an impairment of the ubiquitin-proteasome pathway, the main proteolytic cellular system to degrade damaged proteins [39].

MSC senescence involves multiple signaling pathways such as p53/p21, retinoblastoma (p16/RB), protein kinase B (Akt)/mTOR, mitogen activated protein (p38MAP) kinase and signal transducer and activator of transcription 3 (STAT3), finally causing a permanent cell cycle arrest and irreversible damage [57,61,62,63].

Redox control has a central role in MSC senescence development. At a steady state, MSCs prevalently perform glycolysis and have low ROS levels [64]. However, with progressive replications, a marked production of ROS occurs, inducing damage to cellular macromolecules, with the involvement of several cellular pathways including p53, forkhead box transcription factors of the class O (FOXO1), nuclear factor erythroid 2-related factor (2Nrf2), micro RNAs (miRNAs) and long non-coding RNAs (lncRNAs) (for a comprehensive review, see [65]). Toll-like receptor 3 (TLR3) is upregulated in senescent human umbilical cord MSCs (UC-MSCs) and can drive senescence through the Janus kinase 1 (JAK1) pathway, a key regulator factor in senescent cells. Interestingly, the process is driven by a toll-like receptor 3 (TLR3)-dependent SASP autocrine loop where interferon β (IFN-β) upregulates in turn TLR3 [66]. The conserved small nuclear non-coding RNA Rn7SK is overexpressed in senescent adipose-derived MSCs (ASCs) and, conversely, its transient knockdown leads to delayed senescence [67].

### 3.1. DNA Damage

DNA damage is one of the key drivers of aging and there is strong evidence that age-related accumulation of DNA damage in MSCs is partly responsible for age-related cellular dysfunctions [23]. Signals generated downstream DNA damage are mostly addressed to counteract transformation but can also promote tissue dysfunction. A variety of mechanisms can contribute to DNA damage accumulation in MSC aging: telomere shortening, replicative stress and reduced DNA repair system efficiency [18,68]. Accumulation of DNA damage can lead to genomic instability and ultimately affect differentiation ability, self-renewal ability or induce cell transformation [2]. DNA lesions trigger ataxia telangiectasia mutated (ATM) and ataxia telangiectasia and Rad3-related protein (ATR) pathways and checkpoint activation, inducing in turn cell cycle arrest (to allow for damage repair) or, conversely, senescence and apoptosis (to avoid replication of damaged cells in case of too high levels of DNA damage). While the ATM pathway is mostly involved in double strand break (DSB) repair [69], the ATR one is prevalently involved in single strand break (SSB) and replicative stress-derived damage repair [70]. Murine bone marrow MSCs (BM-MSCs) in long-term expansion progressively lose their ability to recognize DSBs (as detected by phosphorylated histone H2AX-γH2AX- and 53BP1 foci); they show a slower repair kinetics and a consequent increased number of residual DSBs, due to an impaired ATM-mediated DNA damage response [68].

### 3.2. Chromatin Remodelling

One of the characteristics of aged MSCs is a general chromatin reorganization. MSCs have a peculiar chromatin architecture correlated with their uncommitment, multipotency and self-renewal. Heterochromatin domains (chromatin regions structurally inaccessible and usually transcriptionally inactive) are critical for gene expression regulation, DNA repair and maintenance of genomic stability. They are progressively lost during aging, whereas heterochromatin reestablishment promotes longevity [71,72]. Interestingly, DiGeorge syndrome critical region 8 (DGCR8, a critical component of the canonical microprocessor complex for miRNA biogenesis, downregulated in naturally and pathologically aged MSCs) was found to play a role in heterochromatin organization maintenance and aging attenuation, thus emerging as a possible therapeutic target for age-related disorders. This DGCR8 activity is miRNA processing-independent and it is played through interaction with nuclear Lamin B1 and other heterochromatin-associated proteins [73]. A role in heterochromatin reorganization in relationship to MSC aging was also described for lysine-K- specific demethylase 3A (KDM3A) and lysine-K- specific demethylase 4C (KDM4C) enzymes, two H3K9 demethylases. They activate condensing components during MSC senescence and inversely correlated with aging. Their suppression induces a strong DNA damage response and aggravates cellular senescence, whereas their upregulation blocks DNA damage response, thus highlighting a protective role of these enzymes as a surveillance mechanism to limit DNA damage accumulation [43]. As multifunctional modulator of gene expression with the ability to regulate chromatin architecture, methyl-CpG binding protein 2 (MECP2) has a key role in stem cell function and aging. In a mouse model of Rett Syndrome, impaired MECP2 expression is associated to increased senescence and accumulation of DNA damage, through RB2/P130-p16 pathway activation and without impairment of the autophagic process [60].

Deregulation of the chromatin remodeling complex switch/sucrose non-fermentable (SWI/SNF) through Brahma-related gene 1 (BRG1, an ATPase subunit of the complex) can trigger MSC senescence by suppressing homeobox transcription factor (NANOG) transcription and by modulating the expression of proteins involved in nuclear architecture, suggesting a role of BRG1 as a global regulator of gene expression [74]. 

### 3.3. Epigenetics

Epigenetics (heritable changes in gene expression without DNA sequence alterations) include DNA methylation, histone modifications, chromatin remodelling, all influencing chromatin architecture and in turn gene expression. Epigenetics has a fundamental role in stem cell biology (reviewed by [75]), including stem cell senescence. Recent evidences highlighted that epigenetic regulation is involved in MSC senescence [2,75,76,77]. A comprehensive evaluation of the dynamic of epigenetic changes and its effects on stemness and senescence is still missing [75]. Lu et al. [78] showed that octamer-binding transcription factor 4 (OCT4) maintains the self-renewal ability of human hair follicle MSCs and reverses senescence by suppressing the expression of p21 through the upregulation of DNMTs (DNA methyltransferases) leading to an overmethylation of p21 promoter and thus inhibiting its transcription and activity. Epigenetic dysregulation of histone H3 acetylation in K9 and K14 in the promoter regions of stemness genes OCT4 and sex determining region Y-box 2 (SOX2) plays a key role in MSC aging [77]. Moreover, inhibition of histone deacetylase (HDAC) function promotes apoptosis and senescence in human MSCs [75]. General DNA methylation levels appear to decrease with MSC aging and this is associated to DNMT downregulation [75]. DNMT1 and 3b inhibition with specific siRNA (short interfering RNA) or 5-azacytidine, induced senescence in UC-MSCs and p16 and p21 upregulation. Interestingly, DNMT inhibition changed histone marks in active form and induced CpG island demethylation in p16 and p21 promoter regions. It also downregulated polycomb proteins and upregulated miRNAs targeting polycomb proteins expression levels [79,80]. All these data point to a critical role of DNMTs and HDACs in regulating MSC senescence. Through molecular profiling, the role of epigenetic factors (including miRNAs) in cellular senescence has been studied [45,47,81,82]. One of the most interesting recent findings is the association of aging to very reproducible DNA methylation patterns. A specific CpG methylation pattern can therefore represent a biomarker to estimate chronological age, thus functioning as an “epigenetic clock” [83]. In a similar way, regulated and reproducible DNA methylation changes occur during in vitro replicative senescence and can be used to monitor senescence of cell preparations [81,82,84]. Another epigenetic modification of DNA bases is represented by 5-hydroxymethylcytosine (5hmC), recently proposed as additional epigenetic age-related biomarker, associated to 5 methylcytosine (5mC) loss [85].

### 3.4. Autophagy

Recent studies indicated that autophagy is required to maintain stemness and differentiation capacity of stem cells and that autophagy is impaired in stem cell aging (reviewed by [55]). This deficiency can be restored by rapamycin and spermidine treatment [86,87]. Correct functioning of autophagy has also the potential to preserve the anti-inflammatory properties of MSCs, that are mainly exerted through their secretome [88], particularly through the released exosomes and microvesicles [89]. Conversely, evidence is accumulating that vesicles released from MSCs have the ability to rescue autophagy in the target cells [90].

It was demonstrated that autophagy plays a role in the maintenance of BM-MSCs during aging: autophagy declines in BM-MSCs and bone cells with age; its activation could alleviate aging of BM-MSCs and restore aged BM-MSC osteogenic differentiation and proliferation. Autophagy activation also partially restores bone loss in mice, via regulating ROS levels and p53 expression. Conversely, autophagy inhibition could cause senescence of young BM-MSCs [91]. Young quiescent MSCs display a constitutive autophagy activity decreasing with aging. The impairment occurring in old cells is related to their lack in autophagosome formation capacity. Moreover, genetic impairment of autophagy in young MSCs promotes the entry into premature senescence [86].

Available data on the relationship between autophagy and senescence are partly contradictory: actually, autophagy may promote or counteract senescence depending on the cellular context and on the kind of stress [39]. Autophagy can protect BM-MSCs from oxidative stress [92] and, conversely, it has been proven to be a requirement for maintenance of replicative senescence of MSCs [93]. A possible interpretation of these contradictory results is that MSCs try to lead with stress by inducing autophagy to remove damaged components thus counteracting aging; however, in case of impaired autophagic flux and therefore of persistent cell damage, autophagy could worsen senescence.

### 3.5. SASP

Cell senescence is a dynamic process that can be influenced by cell to cell communication and signals from the microenvironment, including paracrine/autocrine soluble factors, gap junctions, extracellular vesicles (EVs). EVs include exosomes and microvesicles (MVs) transporting lipids, proteins, miRNAs, delivering signals to other cells via endocytosis and membrane fusion. They are fundamental mediators of MSC activity. Recently, MSC-derived EVs gained much attention for MSC-based therapies as responsible for MSC activity and as possible cell-free alternative to MSC cellular treatments [94]. EV content is not static, but highly influenced by the niche/microenvironment the MSCs are imbedded in [13]. In fact, EVs from BM-MSCs of differently aged donors show significant age-dependent differences in their content and consequently immune profile [95]. MVs from young or old MSCs contain youth or senescence signals, respectively. In a murine model, old hematopoietic stem cells (HSCs) showed restored functionality and rejuvenation after intercellular transfer of MVs from young MSCs, containing higher levels of autophagy-related mRNAs and sirtuins [96].

In an attempt to define common and specific SASP components, Ozcan et al. [32] exposed human ASCs and BM-MSCs to different genotoxic stresses (oxidative, chemical, physical and replicative) and analyzed the resulting cell secretome. By mass spectrometry followed by gene ontology analysis they were able to distinguish four SASP classes (extracellular matrix; cytoskeleton; cell junctions, metabolic processes, ox-redox factors; regulators of gene expression) common among the different stressor conditions. They also found 11 proteins exclusively expressed in all conditions possibly representing a senescence signature [32].

## 4. In Vivo MSC Senescence

MSCs in adult tissues and organs constitute a reservoir of precursor multipotent cells kept in the so-called “niche”, a specialized structure whose microenvironment supports the maintenance of a suitable pool of precursor cells [97]. However, beside such a “plastic potential”, latest evidence indicates that the major function of MSCs in adult tissues is to ensure the maintenance of tissue homeostasis and to exert anti-inflammatory and immunoregulatory activity, provided that conditions related to the host do not hamper their functionality. Increasing evidence has indicated that beside in vitro expansion, MSCs may undergo aging in vivo. This aging may derive from chronological aging of the host, but also from conditions that anticipate aging such as obesity and that are connected with a status of systemic low grade inflammation, called “inflammaging” [49,98]. In vivo, MSC aging is less described than the in vitro one and in vivo tracking of MSC aging has so far been performed only in animal models. MSC aging in vivo is considered partly responsible for tissue and organ functional decline with organismal aging. During in vivo aging, drifts in the clonal composition of stem cell pools occur, possibly influencing the composition and function of tissues [23]. A progressive stem cell exhaustion occurs, with progressively reduced self-renewal potential, finally leading to tissue deterioration [36].

From a mechanistical point of view, in aging skeleton BM-MSCs show progressively decreased expression of the forkhead box protein P1 (FOXP1) gene, able to counteract expression of p16 [79], the archetypical cyclin dependent inhibitor (CKI) associated with senescence, also known to participate in terminal differentiation onset of several cellular lineages [99]. Indeed, MSCs derived from subjects with increasing age have a lower proliferation and differentiation capacity [100]. At the same time, MSCs derived from adipose tissue of obese subjects have lower self-renewal properties because of increased oxidative and metabolic stress. This stress impacts on ASC mitochondria leading to DNA damage, telomere shortening, reduced proliferation and stemness (as evidenced by decreased expression of NANOG, SOX2 and OCT4), increased apoptosis and senescence [101]. Interestingly, differences are emerging in the performance of ASCs isolated from either subcutaneous or visceral fat and from different anatomical sites [102] or as a function of the metabolic status of the subject, with particular reference to obesity. White adipose tissue deregulation is pivotal in metabolic syndrome. By virtue of the M2 to M1 transition of the macrophages resident in the “niche” and the overall inflammatory environment, ASCs in adipose tissue of obese subjects rather than exerting anti-inflammatory activities release inflammatory cytokines, chemokines and adipokines [103] and circulating miRNAs [104]. Some of these miRNAs target proteins in charge of protecting the cells in conditions of metabolic/oxidative stress [105], such as AMPKα (AMP kinase α) and PPARγ (peroxisome proliferator-activated receptor γ). This results in increased DNA damage and senescence in ASCs, thus short-circuiting their stemness [105]. The obese environment is also responsible for a specific DNA methylation signature that impacts on the functionality of the mitochondria, with changes in mitochondrial shape and number and, consequently, on the functional properties of such cells, with regards to adipogenesis, inflammation, and immunosuppression [106]. In keeping with the above described dysfunction of MSCs derived from obese subjects, we recently reported that, in 3-D ASC cultures, the extent of DNA damage as measured by mean of the marker histone H2AX phosphorylated at Ser139 (γH2AX), that is an indirect indication of double strand breaks, is correlated with a score that takes into account both the BMI (body mass index) and the age of the donor [107].

Senescence and inflammation boost each other, because of the increased expression of the SASP that includes inflammatory mediators and matrix degrading enzymes. The SASP strongly impacts on tissue homeostasis and is mainly dependent on NF-κB activation [108].

Mitochondria from senescent cells release a higher amount of ROS but, more importantly, are pivotal in the pro-inflammatory features of senescence [109] and may be targeted to control SASP [110]. Nuclear genotoxic stress arises, at least in part, because of mitochondrial-derived ROS, and this spontaneous DNA damage is able to trigger NF-κB activation [111,112] that sustains oxidative stress and worsens cellular senescence, with a consequent age-related physiological decline. The fundamental role of mitochondria has been confirmed by using Parkin-mediated widespread mitophagy, that was successful to tune both increased ROS and increased SASP [113]. Therefore, homeostatic mechanisms ensuring proper recycle of damaged organelles are of paramount importance for tissue functionality. To keep functional mitochondria, it is therefore useful to increase “mitophagy”, i.e., autophagy of mitochondria. It has been confirmed that MSCs deficient in autophagy-related proteins are more susceptible to oxidative stress and mitochondrial dysfunction [114]. Beside increased mitophagy, MSCs have adopted a particular strategy to accomplish this goal, i.e., they outsource mitophagy to bystander macrophages in the niche, at the same time delivering miRNA to inhibit macrophage activation that may occur since mitochondria may trigger a pattern recognition receptor reactivity [115]. On the other hand, MSCs may sense mitochondria released from damaged somatic cells as a signal to increase their modulatory activity [116].

MSC aging is considered responsible for impaired fracture healing with age [15]. In vivo MSC senescence implies reduced osteogenic capacity, thus contributing to age-related diseases such as osteoporosis. In ASCs, miR-1292 was found to positively regulate cell senescence through the wingless-related integration site (Wnt)/β-catenin signalling pathway and targeting frizzled 4 receptor (FZD4), thus emerging as a potential target to treat osteoporosis [117]. Moreover, Liu et al. [118] demonstrated that the loss of osteogenic potential in aged BM-MSCs is mediated by p53 increase through the miR-17 pathway. MSC aging was also correlated to impaired hematopoietic functions: BM-MSCs (fundamental for the maintenance of the hematopoietic stem cell compartment in the bone marrow) from aged donors were shown to activate a SASP-like program contributing to young HSCs functional impairment by promoting an inflammatory state in HSCs [119]. Senescence of synovial and subchondral BM-MSCs is likely to contribute to joint alteration and osteoarthritis (OA) development: this was shown in vitro and in vivo with p16-positive human MSCs co-cultured with OA chondrocytes or intra-articularly injected in young mice [120]. BM-MSC number was reduced in adulthood compared to childhood together with an increase of senescence markers (ROS, p21, p53) [121]. Consensus is obtained on the age-related reduced osteogenic differentiation ability (in line with the age-dependent loss in bone-forming efficiency) in favour of adipogenic differentiation. This was associated to the decrease of the microRNAs miR-27a54, miR-27b, Let-7G, and miR-106a55, shown to be required for osteogenic differentiation potential maintenance [28,79].

In vivo MSCs are in a semi-quiescent state, therefore replicative exhaustion is unlikely to be a primary cause of MSC senescence in vivo, that is primarily due to microenvironmental and hormonal conditions [28].

Frequently, in vivo aging is indirectly evaluated by comparing in vitro behavior of MSCs from young and old donors. Numerous data were published demonstrating different performances of MSCs from differently aged donors. In general, aged MSCs display senescent features when compared with cells isolated from young donors, concomitant with reduced viability, proliferation and differentiation ability [121,122,123]. In minimally cultured BM-MSCs from differently aged donors, Ganguly et al. [124] observed a reduction in number of CFUs (colony forming units), with increasing donor age, in accordance with others [90,121,125]; colony density was also reduced with age, indicating an age-related decline in number and proliferative capacity of MSCs. Conversely, minimal transcriptional and phenotypic changes were observed [124]. MSCs from foetal tissues are more plastic and grow faster than MSCs from adult bone marrow [126]. Amniotic fluid MSCs (AF-MSCs) are less prone to senescence compared to adult BM-MSCs and more efficiently respond to DNA damage and return to basal conditions [127]. These features were also associated with significantly reduced differentiation potential in aged compared to young MSCs. ASCs from elderly patients are characterized by high oxidative stress levels, increased senescence, impairment of cellular viability and activity [128]. By comparing ASCs from young, adult and aged donors, an age-dependent decrease in number, proliferative potential and differentiation ability was observed together with an increase in senescence levels [122]. Accordingly, Liu et al. [30] described major functional differences among ASCs from differently aged groups of donors, despite a phenotypically similar profile: with increasing donor age, in vitro cultured ASCs showed a decline in CFUs, SVF (stromal vascular fraction) cell yield, differentiation potential, migration ability and proliferation rate; they also showed increased SA-β-gal activity, ROS and p21 levels [30]. UC-MSCs from term umbilical cord vein show stronger immunomodulatory activity than preterm ones [129].

## 5. In Vitro MSC Senescence

MSC aging in vitro is accelerated compared to in vivo aging for the high replicative stress imposed by the culture conditions to cells and possibly for the absence of the protective environment that the stem cell niche creates in vivo. During extensive cultivation MSCs progressively age with a parallel decrease in telomere length, a modified DNA methylation pattern, altered membrane glycerophospholipid and sphingolipid composition [2,130,131]. Phenotypic changes are accompanied by a progressive loss of potency in terms of reduced homing, migration and differentiation ability evidenced in vitro [2,31,132,133,134]. Even if these changes presumably mirror in vivo alterations, at present there is no demonstration that the same alterations occur in vivo in men. Immunomodulatory properties of MSCs are affected by aging; both aged BM-MSCs and ASCs were shown to less efficiently inhibit T cell, but not NK and B cells, proliferation in vitro. This loss of function is associated with a decreased indoleamine 2,3-dioxygenase (IDO) activity in response to inflammatory stimuli, due to IDO degradation by the proteasome [27].

MSC proliferation rate progressively decreases during in vitro culture [26,95] until cells reach a senescent state with growth arrest [21,24]. Cell cycle arrest usually occurs after about 20-30 replications, depending on donor age, tissue source and culture conditions [24]. UC-MSCs cultured until replicative exhaustion (approximately at 49 mean population doublings, mPDL) showed a significant transcriptome drift rapidly accumulating after culture passage P5 (mPDL 27) [135]. DNA damage progressively accumulates with cell aging increasing the risk of genomic instability [2]. Accordingly, in vitro aged BM-MSCs display increased γH2AX [136]. In general, MSC phenotype is stable in culture [17,26,137]; conventional surface markers seem not modified during in vitro aging, whereas Stro-1 (stromal cell surface marker-1), CD106 (VCAM-1) and CD146 (MCAM) appear downregulated during prolonged culture [2,29]. While immunogenic properties are conserved, immunosuppressive properties, on the contrary, are reduced with culture passages [26].

### 5.1. Epigenetics: In Vitro Data 

By BM-MSC genome wide analysis using bisulfite sequencing-based methods, Pasumarthy et al. [138] demonstrated in vitro expansion-induced methylation changes. These changes were enriched at known distal transcription factor binding sites such as enhancer elements, instead of CpG-rich regions, and associated with modified gene expression. Based on these changes, authors constructed methylation regulatory networks and identified putatively abrogated signalling pathways [138]. Choi et al. [139] reported hypermethylation, after long-term expansion, at genes related to MSC senescence, DNA replication, cell-cycle and adipogenic differentiation. Senescence-associated DNA methylation changes are enriched in intergenic and non-promoter regions [47]. With high-resolution genome-wide technologies significant methylation changes outside of transcription start sites were reported in adult mouse tissues, prevalently at introns, enhancers, and regions with lower CpG densities [140,141,142].

By global genomic methylation, Phermthai et al. [143] found epigenetic instability in high passage cultures of AF-MSCs. This instability correlated with loss of differentiation potential. Histone H3/H4 acetylation is significantly reduced with advancing culture passages in association with enhanced global histone deacetylase activity [77,144]. In general, there is a gradual decrease in global DNA methylation with MSC aging in culture [2,85,145]. Hypomethylated regions are preferentially localized in genes related to morphogenesis and prevail over hypermethylated ones, enriched in genes associated with differentiation [76]. In terms of methylation profile, age-related changes occurring in culture overlap those observed in vivo [45], with epigenetic modifications influencing gene expression profile and reducing stemness and MSC total number [76]. This supports the hypothesis that in vitro replicative senescence partially mirrors in vivo aging [15]. An “epigenetic signature” was identified in human BM-MSCs. This signature is represented by 6 CpGs correlating with PD performed in culture [81,146]. However, since MSC preparations are a heterogeneous mix of subpopulations at different aging steps, CpG methylation profiles greatly differ among subpopulations, thus limiting the univocal application of this signature [147]. 

The discoidin domain receptor 2 (DDR2, a tyrosine kinase collagen receptor expressed by MSCs) was found to be correlated with in vitro BM-MSC senescence: it plays a relevant role in BM-MSC commitment towards osteogenic differentiation rather than adipogenic; it progressively decreases with culture passages; its inhibition in low-passage BM-MSC recapitulates senescence features. DDR2 plays therefore a major role in regulating the in vitro senescence of human BM-MSCs [136]. Moreover, coactivator-associated arginine methyltransferase1 (CARM1) was found to be the upstream up-regulator of DDR2 expression via increased H3 methylation, thus contributing to rejuvenation of late passage BM-MSCs [136]. Decreased expression of HDACs was also observed in senescent MSCs, and treatment with HDAC inhibitors has been shown to induce cellular senescence [144]. Moreover, epigenetic dysregulation of histone H3 acetylation is associated to human placental MSC aging and differentiation in long-term cultures [77]. These data point to a relevant role of histone modification in cell senescence. Histone acetylation and deacetylation are catalysed by histone acetyltransferases (HATs) and HDACs. E1A binding protein (p300) is a crucial member of HAT family, acting to facilitate acetylation-mediated transcriptional activation. Data from cultured UC-MSCs suggest that a decline of p300 contributes to aging by activating p53/p21 pathway [148].

Due to the fundamental role emerging for epigenetics in stem cell senescence, increasing interest is dedicated to epigenetic reprogramming to promote self-renewal capabilities of MSCs, increasing their longevity and potency by contrasting senescence induced by culture expansion. 

### 5.2. Autophagy: In Vitro Data

Autophagy and senescence appear to be regulated by overlapping pathways, even if the link between autophagy and senescence is not so clear and contradictory evidences were published. Autophagy is significantly reduced in aged BM-MSCs compared with young BM-MSCs. By inhibiting autophagy, young BM-MSCs turn into a relatively aged state and, conversely, by activating autophagy the biological properties of BM-MSCs are partially restored [91]. SA-β-gal positive BM-MSCs show Rb phosphorylation, mTOR downregulation, increased p16, p21 and p53 levels flanked by autophagy upregulation. This upregulation is alleviated by p53 knockdown, suggesting a role of p53 in this mechanism [149]. Chang et al. [150] demonstrated in BM-MSCs that high glucose induced autophagy correlated to the development of cell senescence. This effect can be reverted by NAC (N-acetylcysteine) treatment, thus indicating that hyperglycemia can induce MSC senescence through an oxidant-mediated autophagy contributing to bone marrow niche dysfunction [150].

On the contrary, other experimental data indicate a reduction in autophagy in senescent cells. Endometrium-derived MSCs enter the premature senescence in response to oxidative stress via activation of the ATM/p53/p21/Rb pathway. While ATM inhibition allows to escape the permanent cell cycle arrest by re-entry into the S phase, p53 suppression shifts senescence towards autophagy [151]. Acute senescence induced by oxidative stress, doxorubicin treatment and X-ray corresponds to a reduction of the autophagic flux [39,152]. 

### 5.3. ROS (Reactive Oxygen Species) Production: In Vitro Data

MSC longevity and functions are affected by oxidative stress. Indeed, increased reactive oxygen species (ROS) inhibit MSC proliferation, drive senescence, enhance adipogenic but reduce osteogenic differentiation, and impair immunomodulation. Cellular aging is associated with augmented ROS production as a result of the aerobic metabolism, inducing DNA damage and senescence. Increase in ROS levels and the resulting DNA damage are strictly related to aging [153]: augmented ROS levels were observed in senescent MSCs together with a decrease in the expression of the antioxidant enzymes catalase and superoxide dismutase (SOD)1 and 2, in association with differentiation ability decline, suggesting that endogenous ROS accumulation plays a key role in stress-induced MSC senescence. This was partly reverted by ascorbic acid treatment [154,155].

Nuclear factor erythroid 2-related factor 2 (NRF2), a fundamental transcription factor acting in response to oxidative stress, with a central role in MSC survival under oxidative damage, declines during MSC senescence, while its activation induces lifespan extension of MSCs [155].

Persistent oxidative stress and DNA damage response activate p53 and p38MAPK pathways, both responsible for human MSC cell cycle arrest after ROS exposure [156].

BMI1, a member of the polycomb repressive complex protein group is upregulated in hypoxic-cultured UC-MSCs. Its knock-down induces a decrease in cell proliferative and immunomodulatory abilities, along with cyclooxygenase-2 (COX-2)/ prostaglandin E2 (PGE2) downregulation and MKP-1 (mitogen-activated protein kinase phosphatase-1) suppression [157]. Overexpression of BMI1 in MSCs exerts antiaging and antiosteoporosis effects by inactivating p16/p19 signaling and inhibiting oxidative stress [158].

Mitochondrial dysfunction and oxidative stress affect MSC metabolism and function. This is true also in vitro, as evidenced by lower ROS levels and lower mitochondrial potential and activity in MSCs with higher expansion potential and higher CFUs compared to MSCs with shorter in vitro life and lower CFUs [159]. 

Contradictory results have been published concerning senescent MSC metabolism; Capasso et al. [39] documented a shift from glycolysis to oxidative phosphorylation, whereas Fernandez-Rebollo et al. [160] observed a metabolic switch from oxidative to glycolytic pathways, possibly to avoid further damage by ROS.

Modulation of sirtuin expression and activity may represent a method to reduce oxidative stress in MSCs [161]. Altered mitochondrial ROS homeostasis is a pivotal factor in cell senescence induction. Mitochondrial ROS levels are controlled by the mitochondrial localized superoxide dismutase (MnSOD) 2, whose activity is principally regulated by Sirtuin 3 deacetylase [162]. Sirtuins (Sirt) are NAD-dependent protein deacetylases involved in aging, oxidative stress, and metabolism. Sirt3 (the principal mitochondrial deacetylase involved in reducing oxidative stress) expression level plays an essential role in delaying MSC cellular senescence, as it is involved in mitochondrial homeostasis maintenance and oxidative stress regulation [163]. Sirt3 decreases during MSC expansion in vitro and, conversely, its overexpression in late passage MSCs reduces senescence, oxidative stress, and enhances differentiation ability [164]. Severe oxidative stress reduces Sirt3 levels in young human MSCs and, conversely, Sirt3 overexpression, by activating MnSOD and catalase (CAT), protects human MSCs from apoptosis under stress. In normal conditions, Sirt3, MnSOD and CAT levels, as well as cell survival, are similar in young and old MSCs, whereas in stress conditions older cells show a significantly reduced capacity to manage oxidative stress [163].

Ephrins, a family of proteins serving as ligands of the eph receptor (a tyrosine kinase receptor), have been involved in several cellular functions. EphrinB and EphB signalling involve the regulation of the migration, proliferation and immunomodulation of MSCs. Jung et al. [162] correlated the down-regulation of EphB2 and the up-regulation of EphrinB2 to progressive in vitro senescence of UC-MSCs. Conversely, EphB2 signalling activation induces Nrf-2 nuclear translocation and Sirt3 upregulation thus contributing to mitochondrial ROS scavenging and maintaining cell replicative capacity. Sirt1 expression and activity decreases with age. Selective knockdown of Sirt1 (a NAD-dependent histone deacetylase) in human MSCs at early passages slows down cell growth and accelerates cellular senescence. Conversely, overexpression of Sirt1 delays senescence, p16 accumulation, and induces maintenance of adipogenic and osteogenic potential. In addition, we found that the delayed accumulation of the protein p16 is included among the effects of Sirt1 [165]. ASCs from elderly donors show reduced differentiation ability into Beige adipocytes (brown-like adipocytes) compared to children ASCs, together with an impaired expression of Sirt1 and Sirt3 that contributes to senescence promotion. Conversely, Sirt1 overexpression impairs the p53/p21 pathway, thus counteracting senescence and restoring differentiation ability [166]. FOXQ1 binds directly to the Sirt1 promoter to regulate cellular senescence and migration; its overexpression in human UC-MSCs counteracts senescence through enhancement of cell proliferation and viability, reduction of p16, p21, and p53, and promotion of PCNA (proliferating cell nuclear antigen) [167].

### 5.4. Telomeres: In Vitro Data

Adult stem cells are characterised by longer telomeres compared to mature cells from the same tissue [168]. MSCs display absent or low telomerase activity [2,29,169] therefore their telomeres progressively shorten at each cell division [28,29,145,170,171]. It is known that excessively short or altered telomeres induce genomic instability and are recognized as a DNA damage signal. However, experimental data show that in vitro telomere shortening does not affect cell cultures until several passages are performed [2,26,29].

In accordance to progressive telomere shortening, telomerization of human MSCs through hTERT (human telomerase reverse transcriptase) overexpression allows to bypass the replicative senescence phenotype and to improve in vitro and in vivo functions, while maintaining the cellular phenotype [172]. This was also observed by Trachana et al. [173] by exposing MSCs overexpressing hTERT to acute oxidative stress. Cells overexpressing hTERT showed lower DNA damage and higher SOD and catalase activity as compared to control cells of the same passages, indicating hTERT as an enhancer of the cellular antioxidant machinery [173].

### 5.5. SASP: In Vitro Data

Late passage UC-MSCs and BM-MSCs secrete higher levels of MVs with smaller size and decreased amounts of highly expressed miRNAs compared to early passage cells. Interestingly, age-related miR-146a-5p increase and CD105 decrease in late passage MVs mirrored the levels observed in the parental MSCs, thus suggesting MV content as a possible biomarker to monitor MSC senescence [174]. Aging decreases the expression of miR-17 (inhibitor of Beclin-1 and Atg 7 expression) in the MSCs, but it increases the partitioning of both miR-17 and miR-34a (inhibitor of Sirt-1 expression) into their exosomes, through age-mediated activation of AKT. Accordingly, AKT signaling inhibition in aged MSCs restores their rejuvenating capacity by increasing the level of autophagy-related mRNAs in their MVs and reducing miR-17 and miR-34a levels in their exosomes [96]. SASP content can be controlled through miRNA manipulation: secretion of the regenerative growth differentiation factor 6 (Gdf6) is regulated by miR-17, whose expression declines with age [175]. During MSC aging SASP is upregulated by GATA4 via monocyte chemoattractant protein-1 (MCP-1) [50]. SASP induces a paracrine mechanism of premature senescence in young cells [176].

MVs dynamically change in relationship to the MSC status. The microRNA miR-146a-5p appeared up-regulated and most of its target genes were down-regulated in both MSCs and MSC-MVs during senescence, suggesting miR-146a-5p as a potential senescent biomarker [174].

The important role played by EVs in cell-cell communication was involved in the loss of bone stem cell population with age. In particular, skeletal muscle EVs carrying senescence-associated miRNAs (miR-34a) can affect BM-MSC viability, decrease Sirt-1 expression and induce BM-MSC senescence, thus suggesting a role of skeletal muscle in age-related decline of BM-MSCs [177]. Conversely, aged MSCs, showing activated AKT signalling which affects EV RNA profile, produce EVs able to cause aging of HSCs [96].

Tunneling nanotubes (TNTs) are thin protrusions of actin microfilaments able to transfer cytoplasm and organelles between connected cells. In MSC spheroids it was demonstrated that TNTs, by bridging cells, can shuttle components from low-passage to high passage MSCs thus modulating senescence [178].

Beside the general detrimental effect of MSC senescence on cell replicative potential and potency, a possible role of senescent MSCs in tumour promotion through SASP components was suggested by several interesting papers. Senescent MSCs were shown to promote tumor development in experimental models thus highlighting the need to reduce as much as possible expansion times and to apply particular care when MSC preparations are administered to cancer patients or to patients with a cancer history. Contradictory results have been published concerning SASP effects on cancer cells: in some cases, cancer cell growth is stimulated, in other cases it is blocked. These different results could be due to the effect of cancer cells on MSCs. Actually, the SASP of naïve senescent cells (not primed by cancer cells) may block the proliferation and induce senescence of an immortalized lymphoblastoid cell line, whereas primed senescent MSCs show an impaired anti-proliferative and pro-senescence activity due to a significant modification of the SASP composition induced by priming with cancer cells [179]. Moreover, treatment of immortalized prostate and metastatic prostatic cancer cell lines with the SASP from naïve acute senescent MSCs induced senescence of immortalized cells but not of cancer cells [180].

Several mechanisms have been proposed for MSC tumor promotion, including immunosuppression, promotion of angiogenesis, contribution to the tumor microenvironment, inhibition of apoptosis and promotion of metastasis [181]. Tumor promotion is exerted through the release of SASP mediators in the tumor microenvironment that stimulates cancer cell proliferation and migration [32,182].

After senescence acquisition, MSCs shift their activity to promotion of resident tumor cells. UC-MSCs induced to senescence by oxidative or replicative stress were shown to promote breast cancer cell proliferation and migration in vitro and in vivo, through IL6/STAT-3 pathway [183]. Tumor promotion can also be exerted by senescent cell-conditioned medium (CM) alone, as demonstrated for CM from senescent ASCs that promotes tumor cell proliferation possibly by galectin-3 induction [1].

## 6. Detection of Senescent MSCs

Different molecules and biological processes have been proposed for senescence state determination and monitoring: proliferation arrest, telomere attrition, DNA damage, epigenetic, transcriptional and metabolic changes. Features to identify senescent cells: flattened and enlarged morphology; cell cycle arrest; increased SA-β-gal activity; senescence-associated heterochromatin foci (SAHF, specialized heterochromatin domains contributing to silencing of proliferation-promoting genes); altered gene expression (p53, p21, p16 increased expression); shortened or dysfunctional telomeres; SASP and DNA-scars (DNA segments with chromatin alterations reinforcing senescence) [39].

However, specific and univocal markers are still missing and, to be reliable, the evaluation of cell senescence should rely on the in vitro assessment of several markers. Since senescence can affect cell functions, it appears essential to evaluate the percentage of senescent cells in each MSC preparation to be delivered to patients. The identification of reliable senescence markers is therefore a critical, pivotal issue for quality assessment and for in vitro monitoring of MSC preparations before clinical application. Several papers focused on this topic, as reviewed in [184]. Here we only present a summary (Table 1) including the most frequently used analyses to assess senescence. For each technique the relevant use, i.e., which senescence feature it addresses, as well as specific advantages and disadvantages, are indicated. 

Beside the known markers indicated in Table 1, a series of new senescence markers has been proposed. The TRAIL (TNF-related apoptosis-inducing ligand) receptor CD264 was proposed as a marker of BM-MSC cellular age (but not of donor chronological age) since it negatively correlates with proliferation and differentiation potential and parallels p21 expression profile [199].

The angiotensin converting enzyme CD143 was found to be expressed only in adult MSCs [200]. Biran et al. [185] proposed a method to detect senescent cells in tissues by combining flow cytometry and image analysis in order to simultaneously acquire information about beta-galactosidase activity and cellular identity.

Endogenous autofluorescence measured by label-free flow cytometric analysis positively correlates with traditional senescence markers and was proposed as fast and non-invasive tool for real-time quantification of in vitro MSC senescence [201].

F-actin turnover, studied by real-time labelling with a fluorogenic probe, was demonstrated to be age-dependent and to decrease in in vitro aged-MSCs [202].

Exploiting high-throughput screening, Ang et al. [203] identified a senescence-specific fluorescent probe (CyBC9) that accumulates in cell mitochondria, thus allowing for rapid, non-toxic and early detection of senescent MSCs as useful tool to screen clinically intended cell preparations.

For clinical use, potency assays are useful to monitor cell properties predictive of therapeutic efficacy, including evaluation of the effects of aging. An in vitro model of low-density MSC growth was for example recently used to evaluate the effect of age on a series of MSC biophysical properties used as predictors of bioactivity. The findings of the study indicated that MSC age is a predictor of adipogenesis, while cell and nuclear shape are strongly associated to hematopoietic-supportive potency [204].

## 7. MSC Rejuvenating Strategies

The Geroscience Hypothesis [205] states that most of the aging-associated morbidities are sustained by common and interdependent conditions that include: chronic low grade inflammation, macromolecular/organelle dysfunction, stem cell dysfunction and accumulation of senescent cells. 

**Figure 2:**
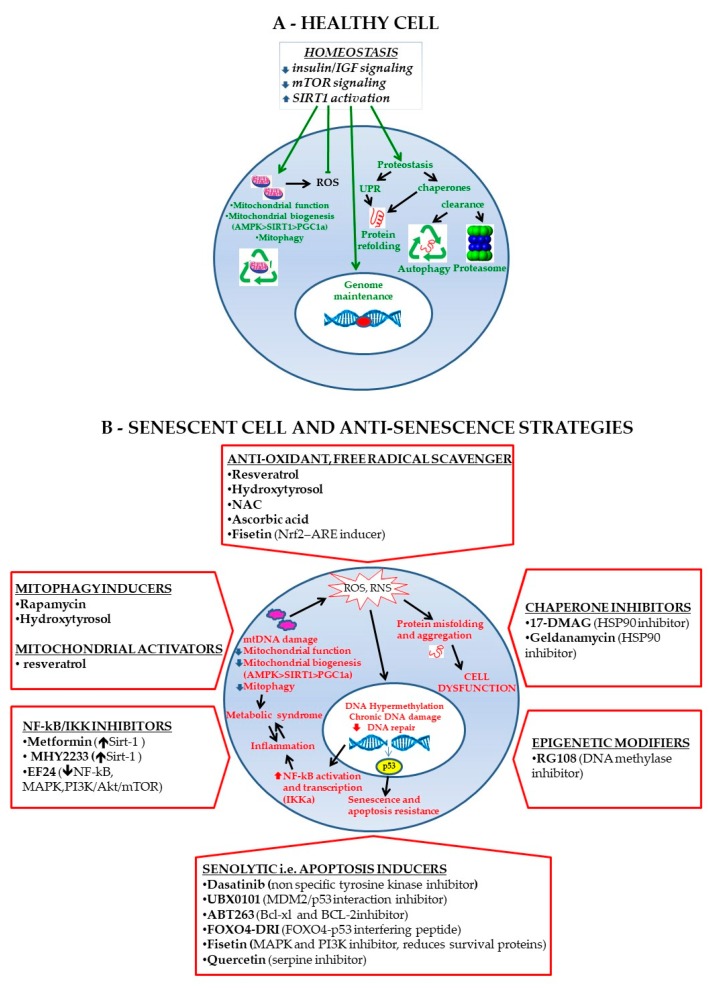
Failure of major homeostatic mechanisms contributes to senescence onset. Failure of major homeostatic mechanisms (autophagy, proteostasis and DNA damage repair networks and mitochondrial respiratory metabolism) in MSC is caused by aging or conditions that accelerate tissue aging, such as the obesity-associated systemic low-grade inflammation that impact on mitochondrial function. (**A**) Homeostasis is tightly controlled by the balance between the insulin/IGF_mTOR signaling and Sirt1. In conditions of homeostasis normal mitochondrial function, biogenesis and autophagy (mitophagy) guarantee that ROS level is kept to the minimum required for intracellular signaling. Both genomic and mitochondrial DNA are preserved from oxidative damage. Proteostasis is guaranteed by the correct functioning of the unfolded protein response (UPR) and clearance via autophagy or the ubiquitin-proteasome system. (**B**) Deranged metabolic factors together with aging contribute to mitochondrial dysfunction, accumulation of ROS (reactive oxygen species) and RNS (reactive nitrogen species) that increase the level of protein misfolding and aggregation, and impact on the integrity of both mitochondrial and genomic DNA. Accumulation of DNA damage cannot be efficiently corrected because mitochondrial dysfunction leads to failure of the energy supply required by the DNA damage response. Persistent DNA damage is responsible for chronic NF-*κ*B activation and inflammation, leading to the “metabolic syndrome.” A positive feedback loop between metabolic syndrome and inflammation is even worsened by excessive ROS and RNS produced by the dysfunctional mitochondria. Persistent DNA damage is then responsible for p53 activation, with functional consequences for the cells that include cell cycle arrest, senescence, or apoptosis, according to an increasing degree. The “senescence pathway” depicted may be targeted for therapeutic intervention: the figure reports the major senotherapeutic classes acting at different functional levels as detailed in the text, although some molecules exert pleiotropic activities.

Targeting one of the above-mentioned conditions has mitigating effects on the other. Hence, the importance of developing methods to reduce MSC senescence [206]. Recent evidences showed that genetic and pharmacological elimination of senescent cells in animal models can extend lifespan and delay the onset of age-related pathologies [207]. Baker et al. [208] demonstrated that the expression of an inducible suicide gene under p16 promoter control can eliminate senescent cells, improve mice health span and age-associated pathologies and delay tumor formation. This was confirmed in other studies selectively ablating senescent cells by genetic systems [208,209] or by drugs (the so-called senolytics) [208,210], thus highlighting that cell senescence contributes to organismal aging. Targeted deletion of senescent cells from cell populations appears to be a suggestive way to counteract age-related tissue decline. The strategies to counteract MSC aging and to deal with senescent MSCs are described in Figure 2 and are mechanistically distinct in “preventive” and “therapeutic” strategies.

As described previously [211,212], therapeutic opportunities comprise both “senomorphic” treatments, i.e., treatments able to reverse senescence, and “senolytic” strategies, i.e., treatments that selectively eliminate senescent cells, exploiting their “Achilles’ heel”, i.e., by targeting pro-survival pathways that are responsible for resistance to apoptosis [213]. Eliminating senescent cells allows to get rid of the SASP and its pro-inflammatory and pro-senescence activity. As reviewed by [29], the usefulness of such strategy has been shown with functional genomic approaches exploiting knock in (hTERT) and knock down (p16) of selected genes. These approaches are not feasible in humans but provided the proof of principle basis for the search of suitable therapeutic strategies, possibly of natural origin. Most of these treatments have been used in somatic cells, but some reports have been recently accumulating that point at their usefulness also with MSCs.

As a first line intervention, the control of oxidative stress and its damaging effects may delay stromal cell senescence. Hence, the usefulness of in vitro or in vivo supplementation with anti-senescence compounds either synthetic or natural, as recently nicely reviewed in [214]. One of these is represented by ascorbic acid (Vitamin C) that is widely used in cell culture, as a first line antioxidant. Beside a direct ROS scavenging activity, ascorbic acid has the ability to attenuate inflammation and many underlying signaling pathways, as nicely reviewed in [153]. MSC senescence has been put in correlation with increased miR-34a that targets Sirt1 thereby worsening cell apoptosis and senescence. Interestingly, these effects can be reversed by treatment with the ROS scavenger N-acetylcysteine (NAC) [215].

In vitro and in vivo treatment with resveratrol was shown to be able to rescue mitochondrial function in BM-MSCs of aged-prone mice via induction of mitofilin, an inner membrane protein that is fundamental for mitochondrial homeostasis [216]. Rescue of mitochondrial function allows for the recovery of osteogenesis, that is hampered towards increased adipogenesis in senescent BM-MSCs [29]. The critical role of mitochondria was recently confirmed by findings of decreased FGF21 (Fibroblast Growth Factor 21) in late passage MSCs that worsens cell senescence via reduced mitochondrial dynamics (increased fusion and decreased fission) and increased ROS production. This is a consequence of reduced AMPK phosphorylation [217], as confirmed by reversal of this phenotype obtained by using an AMPK activator.

Damaged mitochondria may negatively impact on cell function, therefore inducers of autophagy and mitophagy (such as rapamycin) via mTOR inhibition, have proven successful in delaying replicative senescence of BM-MSCs. This is obtained via effective p16 inhibition and NANOG increase [218], despite a certain degree of variability among different donors. 

Proteostasis, i.e., the correct maturation of proteins from synthesis to disposal, has a fundamental role in cell homeostasis. When proteins are not properly folded, the “unfolded protein response” (UPR) takes place in the cells to allow for either protein compensative refolding or for disposal at the proteasome. In both cases, a fundamental role is played by chaperone proteins including heat shock proteins (HSP), stress response proteins in charge for supporting proteostasis in cell stress conditions [211,212]. In the case of senescent cells this activity protects cells from apoptosis. Increase in HSP occurs both in senescent cells and in tumor cells, and here again both cell types share a possible drug target. 17-DMAG was preliminarily selected among a small library of pro-autophagic compounds, tested using murine embryonic fibroblasts with reduced DNA repair capacity, which rapidly senesce at atmospheric oxygen. 17-DMAG proved to be an inhibitor of the HSP90 chaperone family with significant senolytic activity in mouse and human cells in vitro, and also able to reduce age-related symptoms in progeroid mice [211].

Many treatments are targeted to increase anti-inflammatory activities in the cells. Probably the most studied compound is metformin. Metformin exerts several pleiotropic activities being able to target a number of aging-associated pathways, including mTOR and NF-κB, via induction of Sirt1. MHY2233 is a resveratrol analogue that is able to activate Sirt1 and its downstream protective effects, including deacetylase activity. In endothelial progenitor cells, MHY2233 was shown to decrease acetylated p53 and downstream p21 expression, and to increase expression of FOXO3a, a transcription factor that enhances Sirt1 transcription and leads to decreased p16 expression [197]. The curcumin analogue EF24, that also possesses senolytic activities as described thereafter, has the ability to counteract major inflammatory pathways [219]. 

Among the epigenetic modifiers, two papers have shown the ability of RG108, a DNA methyltransferase inhibitor, to rescue stemness of BM-MSCs by increasing expression of the mesenchymal markers CD105, NANOG and Sox4, and of TERT, specifically reducing methylation of the promoters [220,221]. At the same time, this treatment was able to reduce senescence markers (p21 and p53) [220].

As extensively reviewed [206], senolytic drugs are molecules able to target one out of five senescent cell antiapoptotic pathways (SCAPs) whose upregulation is responsible for apoptosis resistance of senescent cells, as also confirmed with RNA interference studies. To these five SCAPs that are specifically linked to overexpression of antiapoptotic resistance proteins, a sixth has been added, that refers to an increased UPR and increased level of chaperone proteins, such as HSPs [206]. This feature is shared by cancer cells, and this is the basis for the use of some antitumoral drugs to control cell senescence. There is a substantial cell specificity in the use of such SCAPs, therefore each potential senolytic drug must be tested against the cell type of interest and, eventually, combination of multiple senolytics may also improve effectiveness.

ABT263 (navitoclax) resulted to be the more effective compound in reducing senescence of human BM-MSCs in vitro compared to quercetin or other senolytics [222]. Similar results were obtained with murine mesenchymal osteoblast progenitors, where ABT263 proved to be able to reduce age-dependent changes in cells derived from old mice [223]. In keeping with the above findings, ABT263 delivered to mice exposed to total body irradiation was able to clear senescent cells [224] including senescent HSCs and senescent muscle stem cells. A senolytic treatment of dasatinib (anti-cancer drug) plus quercetin (natural polyphenol) was instead able to suppress senescence in oligodendrocyte progenitor cells exposed to amyloid β plaque, and the in vivo treatment delivered to mice affected by Alzeheimer’s disease was able to ameliorate neuroinflammation and cognitive deficits [225]. Similar findings were obtained in vivo using a mouse model where obesity was induced in order to investigate the role of senescence in obesity-related neuropsychiatric dysfunction. Obesity led to accumulation of senescent glial cells close to the lateral ventricle, the region where neurogenesis takes place in the adult. Treatment with dasatinib plus quercetin led to depletion of senescent glial cells and rescued neurogenesis and mitigated psychiatric-related alterations [226]. In a recent report of the use of dasatinib plus quercetin to treat the diabetic kidney, effective reduction of senescence (as assessed by counting p16 and p21 positive cells) in adipose derived stem cells was evident as early as after 11 days of treatment [227].

Other strategies to counteract MSC senescence were proposed. Expansion limitations of MSCs can be circumvented by cell reprogramming to iPSCs [228,229]. The similarity of MSCs differentiated from iPSCs (or iMSCs) with primary MSCs in terms of immunophenotype, differentiation ability and gene expression profile was demonstrated [228,230]. Most interestingly, senescence associated DNA methylation patterns were cleared during reprogramming reverting cells to a younger state, suggesting iMSCs rejuvenation compared to parental MSCs [228]. The evidence that “full” cellular reprogramming is accompanied by complete dedifferentiation and by a risk of teratoma formation in vivo, led to the choice of a “partial” reprogramming through cyclic induction with pluripotency factors and without full conversion to pluripotency. However, this process is also associated with erasure of cell-type and tissue-specific epigenetic characteristics that are not recapitulated upon re-differentiation towards MSCs. Moreover, premature reprogramming termination does not result in rejuvenation of MSCs and harbours the risk of transformation. This approach is therefore not suitable to rejuvenate cells for cellular therapy [231]. Foetal and old donor iMSCs showed a similar secretome with a rejuvenation-associated gene signature. While MSCs from differently aged backgrounds have different gene signatures, iMSCs, irrespective of donor age, display similar paracrine features, the most relevant characteristic influencing the clinical effect of these cells [229].

Hypoxia, a culture condition more similar to oxygen levels in vivo, was also proposed to counteract cellular senescence. The anti-aging effect of hypoxia seems to be due to increased genomic stability [232] obtained through downregulation of p16 [233] and p21 [234] and upregulation of DNA repair genes. In human placental MSCs cultured in hypoxic conditions, the aging inducer aminoacyl-tRNA synthetase- interacting multifunctional protein 3 (AIMP3) was dramatically repressed with concurrent p16 downregulation. Moreover, AIMP3 enhances mitochondrial respiration and suppresses autophagy, while hypoxia-inducible factor 1α (HIF1α) inhibits AIMP3 [235]. Human urine stem cells (USCs), dental pulp stem cells (DPSCs), amniotic fluid stem cells (AFSCs), and BM-MSCs show enhanced cell proliferation rate, retention of stem cell properties, inhibition of senescence (as indicated by reduced SA-β-gal staining), and increased differentiation ability in hypoxic conditions compared to normoxia [236]. Accordingly, hypoxic-cultured human UC-MSCs showed enhanced proliferation and increased immunosuppressive effects on mitogen-induced mononuclear cell proliferation [157].

A series of antioxidant agents has been proposed as anti-aging additives for stem cell cultures since oxidative stress reduction is expected to improve the efficacy of MSC therapeutic preparations. Among them, the milk protein lactoferrin, a multifunctional iron-binding glycoprotein. MSC treatment with lactoferrin efficiently lowered the levels of hydrogen peroxide-induced intracellular ROS, and suppressed H_2_O_2_-induced senescence and apoptosis via inhibition of caspase-3 and Akt activation [237]. Human ASCs from elderly donors, after treatment with the DNMT-inhibitor 5-Azacytidine (5-AZA) displayed these changes: reversed aged phenotype; increased proliferation rate, decreased ROS levels, increased SOD activity, and increased BCL-2/BAX (B cell lymphoma 2/Bcl-2 associated X protein) ratio, while the accumulation of oxidative stress factors and DNA methylation status were decreased. Simultaneously, mRNA levels of ten-eleven translocation (TET) proteins, involved in demethylation processes, were elevated [128]. 5-Methoxytryptophan (5-MTP), a tryptophan metabolite which controls stress induced inflammatory signals, was shown to protect MSCs from stress or H_2_O_2_-induced senescence. Indeed, 5-MTP treatment of BM-MSCs reduced p16 and p21 expression, SA-β-Gal and IL-6 secretion and increased BrdU incorporation, via FOXO3a and mTOR upregulation [238]. The plant polyphenol resveratrol administered in culture is able to retain proliferative and differentiation abilities of BM-MSCs over multiple passages [239].

In vivo and in vitro overexpression of FOXQ1 in human UC-MSCs resulted in enhanced cell proliferation and viability, reduced expression of markers positively associated with senescence (p16, p21, p53), and increased expression of markers negatively associated with senescence (Sirt1, PCNA) [167], suggesting FOXQ1 as a possible target to optimize stem cell therapy.

The polysaccharide hyaluronan coated on cell culture surfaces was found to maintain quiescence and stemness, to preserve replicative potential, to stimulate telomerase activity and to delay aging of human placental MSCs [240].

MSC cultures are a mix of subpopulations therefore they are heterogeneous in terms of senescence, differentiation potential and functional properties. Old BM-MSC subpopulations not only show impaired functionality, but release factors suppressing the youthful of non-senescent subpopulations thus inducing a functional decline of the whole culture. The selective isolation of youthful subpopulations (based on cell size and SSEA-4, stage-specific embryonic antigen-4, expression) and their expansion in a young microenvironment (conditioned medium from young MSCs) demonstrate the possibility to rejuvenate MSCs from elderly subjects for autologous treatments [241].

## 8. Concluding Remarks

MSCs are fundamental for tissue homeostasis to delay onset of age-related diseases. They also hold widespread appeal for regenerative medicine applications that frequently require MSC in vitro expansion to achieve clinically relevant cell numbers, thus exposing cells to replicative senescence. MSC senescence, occurring both in vivo and in vitro, can strongly affect cell properties with important clinical and safety implications. Therefore, a deep understanding of cellular mechanisms involved in MSC senescence and the set-up of standardized and universal methods to assess and monitor MSC senescence by defining reliable markers appear critical issues. The recent identification of a series of senolytic and senomorphic agents able to clear senescent cells or to revert senescent phenotypes is of particular interest and holds enormous potential for the treatment of age-related pathologies and for MSC cell preparation rejuvenation, thus allowing to overcome expansion limitations.

## Figures and Tables

**Table 1:** Markers of MSC senescence and techniques most frequently used for their detection.

Senescence Marker			Techniques for Detection	Senescent Features, Pros and Cons
Cell morphology	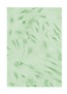	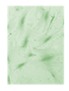	Microscopy [185] Flow cytometry [186] (FSC for size, SSC for granularity)	Senescent MSCs show enlarged and granular cell morphology. Microscopic assessment is easy but only qualitative. Flow cytometric assessment is quantitative
CFU	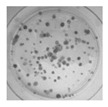		Colony formation in agar culture [187]	The CFU number is a measure of cell clonogeneity and decreases with MSC age. CFU assessment requires careful plating at low density.
Sa-β-gal	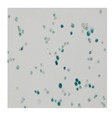		Microscopy (colorimetric activity assay with X-gal) [188] Flow cytometry (fluorimetric activity assay with C_12_FDG) [189] IHC, IF or WB with specific Abs (protein expression) [190]	Senescent cells at low density express a lysosomal β-galactosidase active at pH 6.0, detectable either with activity assays or with a specific antibody. The activity assays can give altered results on high density cultures [191]
8-oxo-dG	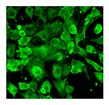		IHC, IF, ELISA [192], HPLC [192]-MS/MS	8-oxodG is a DNA base derivative, robust marker of oxidative DNA and RNA damage
γH2AX	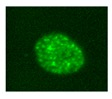 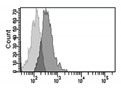 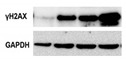		IF Flow cytometry [193] WB	Histone H2AX phosphorylation is an indirect measure of DNA double strand breaks due to physical, chemical, oxidative stress. It indicates that cells organize a DNA damage response, but its persistence sustains senescence of the cells.
Telomeres	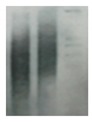 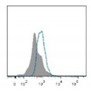		Southern blotting [170] Flow FISH [194] Real-time PCR [194] STELA [195]	Telomere attrition is directly correlated to replicative senescence, but it also occurs after exposure to oxidative damage. The subpopulation heterogeneity must be taken into account and may be addressed with emerging techniques such as STELA, detecting individual telomere length.
MSI	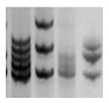		PCR followed by gel or capillary electrophoresis [196]	Repeated sequences variations are an indirect indication of genomic instability and deficient DNA repair due to replicative or oxidative stress. They increase with cell aging.
Gene expression of senescent markers at mRNA level	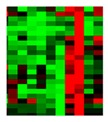		Real-time RT-PCR [33] Microarray RNAseq	Expression of genes related to senescence. Several pathways can be analyzed, but gene expression analysis prevalently focuses on p53 and cyclin dependent kinase inhibitors (p16 and p21)
Expression of senescent markers at protein level	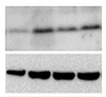 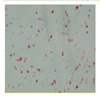		WB [197] IHC IF Flow cytometry	Evaluation of the expression levels of proteins related to senescence (p16, p21, p53, etc.)
Global methylation	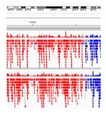		NGS after bisulfite treatment [198]	Genome wide analysis of methylated cytosines.

FSC: forward scatter; SSC: side scatter; CFU: colony forming unit; X-gal: galactosidase substrate; C_12_FDG: fluorogenic galactosidase substrate; IHC: immunohistochemistry; IF: immunofluorescence; WB: western blotting; ELISA: enzyme-linked immunosorbent assay; HPLC-MS: high performance liquid chromatography-mass spectrometry; 8-oxo-gG: 8-Dihydro-8-oxo-2’-deoxyguanosine; γH2AX: phosphorylated H2A histone family member X; FISH: fluorescence in situ hybridization; STELA: single telomere length analysis; PCR: polymerase chain reaction; RT-PCR: reverse transcriptase PCR; NGS: next generation sequencing.
